# Predictive value of lymphocyte subsets and lymphocyte-to-monocyte ratio in assessing the efficacy of neoadjuvant therapy in breast cancer

**DOI:** 10.1038/s41598-024-61632-z

**Published:** 2024-06-04

**Authors:** Hao Zhang, Yan Li, Ya-Wen Liu, Ye-Gang Liu, Xin Chen

**Affiliations:** 1 Department of Breast and Thyroid Surgery, The First Affiliated Hospital of Chongqing Medical University, Chongqing, China; 2 School of Public Health, Chongqing Medical University, Chongqing, China; 3 Department of General Surgery, People’s Hospital of Tongzi County, Zunyi, Guizhou Province China

**Keywords:** Lymphocyte subsets, Lymphocyte-to-monocyte ratio, Neoadjuvant therapy, Prediction, Efficacy, Breast cancer, Breast cancer, Tumour immunology, Risk factors, Inflammation, Lymphocytes, Tumour immunology, Predictive markers, Cancer

## Abstract

Lymphocyte subsets are the most intuitive expression of the body’s immune ability, and the lymphocyte-to-monocyte ratio (LMR) also clearly reflect the degree of chronic inflammation activity. The purpose of this study is to investigate their predictive value of lymphocyte subsets and LMR to neoadjuvant therapy (NAT) efficacy in breast cancer patients. In this study, lymphocyte subsets and LMR were compared between breast cancer patients (n = 70) and benign breast tumor female populations (n = 48). Breast cancer patients were treated with NAT, and the chemotherapy response of the breast was evaluated using established criteria. The differences in lymphocyte subsets and LMR were also compared between pathological complete response (pCR) and non-pCR patients before and after NAT. Finally, data were analyzed using SPSS. The analytical results demonstrated that breast cancer patients showed significantly lower levels of CD3 + T cells, CD4 + T cells, CD4 + /CD8 + ratio, NK cells, and LMR compared to benign breast tumor women (*P* < 0.05). Among breast cancer patients, those who achieved pCR had higher levels of CD4 + T cells, NK cells, and LMR before NAT (*P* < 0.05). NAT increased CD4 + /CD8 + ratio and decreased CD8 + T cells in pCR patients (*P* < 0.05). Additionally, both pCR and non-pCR patients exhibited an increase in CD3 + T cells and CD4 + T cells after treatment, but the increase was significantly higher in pCR patients (*P* < 0.05). Conversely, both pCR and non-pCR patients experienced a decrease in LMR after treatment. However, this decrease was significantly lower in pCR patients (*P* < 0.05). These indicators demonstrated their predictive value for therapeutic efficacy. In conclusion, breast cancer patients experience tumor-related immunosuppression and high chronic inflammation response. But this phenomenon can be reversed to varying degrees by NAT. It has been found that lymphocyte subsets and LMR have good predictive value for pCR. Therefore, these markers can be utilized to identify individuals who are insensitive to NAT early on, enabling the adjustment of treatment plans and achieving precise breast cancer treatment.

## Introduction

Metastasis is the main cause of death in breast cancer, which is the most common malignant tumor in women and is ranked second in mortality among female malignancies^1^. Currently, neoadjuvant therapy (NAT) is commonly recommended for advanced breast cancer, as it can effectively lower the tumor stage, reduce metastasis, and increase the chances of surgery as well as breast preservation^2^. However, the efficacy of NAT varies from individual to individual, making it challenging for clinicians to determine its effectiveness timely and effectively. If the therapeutic effect cannot be assessed promptly, it may cause delays in surgical opportunities and tumor progression, ultimately reducing patients’ survival rates. Although Miller-Payne grading system can be used as a gold standard for postoperative pathological evaluation, it can only be obtained from postoperative specimens, which has a certain delay. After chemotherapy, tumor cells undergo degeneration, necrosis, and the formation of granulation tissue or scar tissue due to hyperplasia, which occupy the original tumor bed. Therefore, in some cases, the mass may not show a significant reduction in size on imaging examination. Ultrasound and other imaging evaluation methods, although capable of assessing treatment response in a timely manner, may encounter limitations in such cases. Consequently, it is crucial to explore a simpler, more timely, and accurate method for evaluating the therapeutic effect. In the past, researchers have been looking for new predictive markers. The relationship between immune cells and tumor cells has been extensively studied with the deepening of research on tumor immunology. It has been found that there are significant immunosuppression and immune disorders in patients with malignant tumors such as lung cancer, esophageal cancer, gastric cancer, colon cancer and gastric cancer, with decreased levels of CD3 + T cells, CD4 + T cells, CD8 + T cells, NK cells and other immune effector cells^3,4^. Immune dysfunction weakens the body’s ability to recognize mutated cells and tumor cells, allowing them to escape from the killing effect of immune system and metastasize from the primary site. In addition, for the special population of patients with malignant tumors, researchers have discovered the dual effects of chemotherapy on the immune function of the body. On one hand, chemotherapy, as a systemic treatment method, kills tumor cells while also killing some normally functioning immune cells, inhibiting the differentiation of immune cells. On the other hand, chemotherapy indirectly reduces the inhibitory effect of tumor cells on the immune system after killing tumor cells^5,6^. Therefore, we speculate that for individuals sensitive to chemotherapy, the tumor burden of the patient decreases after receiving chemotherapy, leading to weaken tumor-related immunosuppressive effect, which may restore the patient’s immune function. However, for patients who are not sensitive to chemotherapy, there is no significant increase in various immune indicators after treatment, and even a decrease may occur, because chemotherapy not only cannot effectively kill tumor cells but may further damage the immune system. Certain studies suggest that pre-existing immune responses may enhance the effectiveness of traditional chemotherapy drugs. Li et al.^7^ has reported a correlation between lymphocytes and the therapeutic response and prognosis of certain tumor patients receiving NAT. Therefore, we speculate that monitoring the lymphocyte subsets in the body may be a novel approach to assess treatment effectiveness.

In addition, inflammatory cells have been discovered in the majority of solid tumors, including colon, stomach, and liver cancers^8,9^. Tumor-related systemic inflammation is not only an important factor promoting tumorigenesis and progression but also an independent factor affecting prognosis^10,11^. Previous studies has demonstrated a 40%-50% reduction in the risk of colorectal cancer associated with the use of nonsteroidal anti-inflammatory drugs (NSAIDs)^12^. The lymphocyte-to-monocyte ratio (LMR) is one of the evaluation indicators for the degree of systemic inflammatory response^13^. Studies have shown that breast cancer patients with high LMR before NAT have significantly prolonged disease-free survival (DFS), and high LMR patients have lower recurrence rates and better survival rates after NAT^14,15^. After effective chemotherapy, the tumor burden decreases and the tumor-related inflammatory response weakens. Therefore, we speculate that LMR can also be used as an effective predictor of NAT efficacy. Moreover, lymphocyte subsets and LMR can be directly obtained from peripheral venous blood, which has the advantages of simplicity, real-time detection, and low cost. The aim of this study is to further explore the predictive value of lymphocyte subsets and LMR for the efficacy of breast cancer patients receiving NAT, providing reference for clinical treatment planning.

## Data and methods

### General information

Collected from August 2022 to May 2023 in the First Affiliated Hospital of Chongqing Medical University, female patients with breast cancer who underwent NAT were included in the case group. At the same time, a control group consisting of 48 female individuals with histologically confirmed breast fibroadenoma was included in the study. The inclusion criteria for the case group were: (1) female patients aged 18–80 years; (2) no acute or chronic inflammation, other tumors, and autoimmune diseases such as hyperthyroidism and rheumatoid arthritis; (3) complete clinical and postoperative pathological data. The exclusion criteria were: (1) pregnant or breastfeeding patients; (2) prior steroid, immunosuppressive or immunoenhancement drug treatment; (3) prior anti-inflammatory drug treatment; (4) patients with chemotherapy contraindications; (5) patients who were assessed as having no response or tumor progression during treatment and required a change in treatment regimen. According to the inclusion and exclusion criteria, a total of 70 patients were included in the case group, with an average age of 52.73 ± 9.90 years. Among them, 38 (54.3%) were postmenopausal. 6 cases (8.6%) had tumor size ≤ 2 cm, 49 cases (70.0%) had tumor size > 2 cm and ≤ 5 cm, and 15 cases (21.4%) had tumor size > 5 cm. 62 cases (88.6%) had lymph node metastasis. According to the eighth edition of the AJCC TNM staging system, ≤ II stage in 24 cases (34.3%) and ≥ III stage in 46 cases (65.7%). Furthermore, based on the different expression statuses of hormone receptor and HER-2 receptor, the case group patients were divided into hormone receptor-positive group, HER-2 receptor-positive group, and triple-negative group. Patients who were hormone receptor-positive and HER-2 receptor-negative were included in the hormone receptor-positive group, with a total of 21 cases (30.0%); patients who were HER-2 receptor-positive regardless of hormone receptor status were included in the HER-2 receptor-positive group, with a total of 36 cases (51.4%); and patients who were negative for both hormone receptor and HER-2 receptor were included in the triple-negative group, with a total of 13 cases (18.6%). Based on a Ki-67 expression cut-off of ≤ 20%, Ki-67 low expression (≤ 20%) in 21 cases (30.0%), and high expression (> 20%) in 49 cases (70.0%). Out of the total patients, 28 (40.0%) achieved pCR while 42 (60.0%) did not. In the control group of 48 benign breast tumor female subjects, the average age was 49.23 ± 11.34 years. Among them, postmenopausal in 19 cases (39.6%). Tumor size ≤ 2 cm in 12 cases (25.0%), tumor size > 2 cm and ≤ 5 cm in 25 cases (52.1%), and tumor size > 5 cm in 11 cases (22.9%). There were no significant differences in age, menstrual status, and tumor size between the two groups (*P* ≥ 0.05), indicating good comparability between the two groups. This study was approved by the ethics committee of the First Affiliated Hospital of Chongqing Medical University. Data collection began on October 13, 2023.

### Treatment method

Both hormone receptor-positive and triple-negative subtypes patients received the TAC neoadjuvant chemotherapy regimen, which included intravenous infusion of 75 to 80 mg/m2 of docetaxel or 175 mg/m2 of paclitaxel on day 1; intravenous infusion of 90/50 mg/m2 of epirubicin or pirarubicin on day 1; and intravenous infusion of 600 mg/m2 of cyclophosphamide on day 1. And HER-2 receptor-positive group patients received a neoadjuvant therapy regimen that included targeted agents (AC-THP regimen), which was an addition of trastuzumab and pertuzumab to the TAC regimen. Three weeks constituted one cycle, and the patients were evaluated for clinical efficacy every two cycles. Patients either continued with the current chemotherapy regimen if the treatment was effective or underwent a change in chemotherapy regimen or surgery if the treatment was ineffective or the tumor progressed. Surgical treatment was performed two weeks after the completion of the prescribed cycle of NAT.

### Ethical approval

The studies involving human participants were reviewed and approved by the institutional ethics committee of the First Affiliated Hospital of Chongqing Medical University, and all experiments were performed in accordance with relevant guidelines and regulations. As this was a retrospective non-interventional study, written informed consent was not required from participants in accordance with national legislation and institutional requirements, and the application for exempted informed consent for the study has been approved by the ethics committee of the First Affiliated Hospital of Chongqing Medical University.

## Detection method

### Lymphocyte subset detection

Case group patients were drawn peripheral venous blood 2 ml before NAT and after 6 cycles of NAT prior to surgery. With heparin as anticoagulant, 100 μl was taken from each sample, added 20 μl of monoclonal antibodies CD3, CD4, CD8, CD16, CD19, and CD56-fluorescently labeled single clones, and incubated in the dark for 30 min. Then phosphate buffered saline (PBS) 3 ml was added for washing, centrifuged at 1500 r/min for 5 min, and washed again with PBS 1 ml. The proportion of CD3 + T cells, CD3 + CD4 + T cells, CD3 + CD8 + T cells, CD3 + CD4 − CD8 − T cells, CD3 + CD4 + CD8 + T cells, CD3 − CD16 + CD56 + NK cells, CD3 + CD16 + CD56 + NKT cells, and CD19 + B cells was detected by flow cytometry. Monoclonal antibodies and flow cytometers were purchased from BD Biosciences (USA), and the operation was carried out according to the instructions of the reagent. Control group patients were also sampled prior to pathological biopsy, using the same method as the case group.

### Routine blood test

Case group patients were draw 5 ml venous blood before NAT and surgery and put it into an anticoagulant tube for inspection. The centrifuge was set to a speed of 1500 r/min with a radius of 15 cm, spun for 10 min. Mindray BC5180 automated hematology analyzer and supporting reagents were used to detect lymphocyte and monocyte counts. The test was repeated three times, and the average value of the three tests was used as the final result. Collected lymphocyte and monocyte counts data were used to calculate the LMR value. (LMR = lymphocyte count/monocyte count).

### Criteria for efficacy evaluation

NAT efficacy was evaluated using the Miller-Payne grading system for breast cancer treatment response. It was divided into grades 1–5. Grade 5 was considered a pathological complete response (pCR). Pathological efficacy evaluation was completed by a senior pathologist and then reviewed by a higher-level pathologist.

### Statistical method

Data analysis was carried out using SPSS 25.0 statistical software. Quantitative data that meet the normal distribution were expressed as the mean ± standard deviation (x ± s), and the independent sample t-test was used for group comparison. Quantitative data that did not meet the normal distribution were expressed as M (P25, P75), and the Wilcoxon (Mann–Whitney U) test was used for group comparison. Multiple group comparisons were performed using the Kruskal–Wallis test (KW test). Qualitative data were expressed as n (%), and the chi-square test or KW test was used for group comparison. Statistically significant indicators in univariate analysis were included in the multivariate analysis model, and binary logistic regression was used for multivariate analysis. The receiver operating characteristic (ROC) curve was used to evaluate the predictive value of each index for NAT efficacy, and the Youden index was used to determine the optimal cutoff value. When *P* < 0.05, the difference was considered statistically significant.

## Results

### Comparison of lymphocyte subsets and LMR levels in the control and case groups

Univariate analysis results showed that there were no significant differences in age, menstrual status, and tumor size between the two groups (*P* ≥ 0.05). The levels of CD3 + T cells, CD4 + T cells, CD4 + /CD8 + ratio, NK cells, and LMR were lower in the case group compared to the control group, with statistically significant differences (*P* < 0.05). There were no significant differences in CD8 + T cells, CD3 + CD4 − CD8 − T cells, CD3 + CD4 + CD8 + T cells, NKT cells, and CD19 + B cells between the two groups (*P* ≥ 0.05) (Table 1).

**Table 1:** Baseline information table and univariate analysis.

	Total	Benign breast tumor patients	Breast cancer patients	*P*-value
Patients	118	48	70	–
Age	51.32 ± 10.59	49.23 ± 11.34	52.73 ± 9.90	0.080
Menopausal status				0.116
Menopausal	57(48.3%)	19(39.6%)	38(54.3%)	
Non-menopausal	61(51.7%)	29(60.4%)	32(45.7%)	
Tumor size				0.216
≤ 2 cm	18(15.3%)	12(25.0%)	6(8.6%)	
> 2 cm and ≤ 5 cm	74(62.7%)	25(52.1%)	49(70.0%)	
> 5 cm	26(22.0%)	11(22.9%)	15(21.4%)	
CD3 +	72.98 ± 7.84	75.16 ± 5.15	71.49 ± 8.98	**0.006**
CD3 + CD4 +	43.76 ± 7.72	46.13 ± 6.63	42.13 ± 8.03	**0.005**
CD3 + CD8 +	24.24 ± 7.46	23.01 ± 6.37	25.08 ± 8.06	0.139
CD4 + /CD8 +	2.03 ± 0.87	2.23 ± 0.88	1.90 ± 0.84	**0.045**
CD3 + CD4 − CD8 −	5.54 ± 3.99	6.32 ± 4.95	5.01 ± 3.10	0.081
CD3 + CD4 + CD8 +	0.43 ± 0.67	0.52 ± 0.98	0.37 ± 0.32	0.246
CD19 + B	13.39 ± 4.67	12.57 ± 3.79	13.95 ± 5.14	0.115
NK	13.05 ± 5.67	14.10 ± 4.81	12.20 ± 6.17	**0.047**
NKT	3.66 ± 3.29	3.9 ± 3.42	3.49 ± 3.21	0.506
LMR	4.40 ± 1.75	5.37 ± 1.85	3.73 ± 1.34	**0.001**

Values in bold represent statistically significant results (*P* < 0.05).

### Comparison of lymphocyte subsets and LMR levels in the pCR and non-pCR groups before NAT

The levels of CD4 + T cells, NK cells, and LMR were lower in the non-pCR group compared to the pCR group before NAT, with statistically significant differences (*P* < 0.05). There were no significant differences in CD3 + T cells, CD8 + T cells, CD4 + /CD8 + ratio, CD3 + CD4 − CD8 − T cells, CD3 + CD4 + CD8 + T cells, NKT cells, and CD19 + B cells between the two groups (*P* ≥ 0.05) (Table 2).

**Table 2:** The relationship between lymphocyte subsets and LMR before NAT with pCR.

Indicators	Total (n = 70)	pCR group (n = 28)	non-pCR group (n = 42)	*P*-value
CD3 +	71.49 ± 8.98	73.95 ± 7.83	69.85 ± 9.41	0.061
CD3 + CD4 +	42.13 ± 8.03	45.19 ± 8.06	40.09 ± 7.42	**0.008**
CD3 + CD8 +	25.08 ± 8.06	24.63 ± 7.79	25.38 ± 8.32	0.706
CD4 + /CD8 +	1.90 ± 0.84	2.05 ± 0.88	1.80 ± 0.80	0.212
CD3 + CD4 − CD8 −	5.01 ± 3.10	4.86 ± 2.55	5.11 ± 3.45	0.739
CD3 + CD4 + CD8 +	0.37 ± 0.32	0.31 ± 0.21	0.41 ± 0.38	0.228
CD19 + B	13.95 ± 5.14	13.78 ± 4.05	14.07 ± 5.80	0.823
NK	12.20 ± 6.17	14.29 ± 6.66	10.80 ± 5.46	**0.019**
NKT	3.49 ± 3.21	3.24 ± 2.55	3.65 ± 3.61	0.601
LMR	3.73 ± 1.34	4.33 ± 1.33	3.34 ± 1.20	**0.002**

Significant values are in bold.

### Comparison of different clinical features in the pCR and non-pCR groups before NAT

There was a significant difference in Ki-67 expression level between the pCR group and non-pCR group before NAT (*P* < 0.05). However, there were no significant differences in TNM staging, lymph node metastasis, and molecular typing between the two groups (*P* ≥ 0.05) (Table 3).

**Table 3:** The relationship between clinical features with pCR.

Indicators	Total (n = 70)	pCR group (n = 28)	non-pCR group (n = 42)	*P*-value
Age	52.73 ± 9.90	51.68 ± 8.26	53.43 ± 10.90	0.473
Menopausal status				0.557
Menopausal	38 (54.3%)	14 (50.0%)	24 (57.1%)	
Non-menopausal	32 (45.7%)	14 (50.0%)	18 (42.9%)	
TNM Staging				0.475
Phase 0–II	24 (34.3%)	11 (39.3%)	13 (31.0%)	
Phase III–IV	46 (65.7%)	17 (60.7%)	29 (69.0%)	
Presence of lymph node metastasis				0.818
Lymph node metastasis	62 (88.6%)	24 (85.7%)	38 (90.5%)	
No lymph node metastasis	8 (11.4%)	4 (14.3%)	4 (9.5%)	
Molecular typing				0.674
Hormone receptor-positive type	21 (30.0%)	8 (28.6%)	13 (31.0%)	
HER-2 receptor-positive type	36 (51.4%)	16 (57.1%)	20 (47.6%)	
Triple-negative type	13 (18.6%)	4(14.3%)	9 (21.4%)	
Ki-67 level				**0.019**
Low expression (≤ 20%)	21 (30.0%)	4 (14.3%)	17 (40.5%)	
High expression (> 20%)	49 (70.0%)	24 (85.7%)	25 (59.5%)	

Significant values are in bold.

### Comparison of changes in lymphocyte subsets and LMR in the pCR and non-pCR groups after NAT

We denoted the magnitude of change in lymphocyte subsets and LMR before and after NAT as Delta-lymphocyte subsets and Delta-LMR. After NAT, significant differences were observed in the dCD3 + T cells, dCD4 + T cells, dCD8 + T cells, dCD4 + /CD8 + ratio, and dLMR between the non-pCR group and pCR group (*P* < 0.05). In both groups, the levels of CD3 + T cells and CD4 + T cells increased after treatment. However, these increases were significantly higher in the pCR group compared to the non-pCR group. The level of CD8 + T cells decreased in the pCR group after treatment, while it increased in the non-pCR group. The change trend of CD4 + /CD8 + ratio was opposite to that of CD8 + T cells. More specifically, the level of CD4 + /CD8 + ratio increased in the pCR group after treatment, while it decreased in the non-pCR group. Additionally, both groups exhibited a decrease in LMR after NAT, but the decrease was significantly higher in the non-pCR group. However, no significant differences were observed dCD3 + CD4 − CD8 − T cells, dCD3 + CD4 + CD8 + T cells, dNK cells, dNKT cells, and dCD19 + B cells levels between the two groups (*P* ≥ 0.05) (Table 4).

**Table 4:** The relationship between NAT-induced changes in lymphocyte subsets and LMR with pCR.

Indicators	Total (n = 70)	pCR group (n = 28)	non-pCR group (n = 42)	*P*-value
dCD3 +	7.44 ± 9.16	10.25 ± 7.92	5.56 ± 9.54	**0.035**
dCD3 + CD4 +	3.13 ± 10.20	6.70 ± 9.94	0.75 ± 9.78	**0.016**
dCD3 + CD8 +	3.24 ± 8.85	− 0.09 ± 8.77	5.45 ± 8.28	**0.009**
dCD4 + /CD8 +	0.01 ± 0.95	0.46 ± 0.96	− 0.31 ± 0.81	**0.001**
dCD3 + CD4 − CD8 −	0.74 ± 3.48	1.59 ± 3.33	0.18 ± 3.50	0.098
dCD3 + CD4 + CD8 +	0.02 ± 0.32	0.02 ± 0.23	0.02 ± 0.37	0.967
dCD19 + B	− 8.70 ± 5.72	− 9.53 ± 5.46	− 8.14 ± 5.89	0.325
dNK	− 3.48 ± 7.03	− 5.36 ± 7.53	− 2.23 ± 6.47	0.068
dNKT	1.00 ± 3.53	1.28 ± 2.64	0.81 ± 4.04	0.593
dLMR	− 1.22 ± 1.41	− 0.70 ± 1.64	− 1.56 ± 1.12	**0.011**

Significant values are in bold.

### Logistic multivariate analysis of factors influencing NAT efficacy

In the multifactorial logistic regression model, the independent variables included lymphocyte subset indexes and LMR before NAT, which were found to be statistically significant in univariate analysis. After adjusting for the effect of Ki-67 level, the model indicated that CD4 + T cells and LMR before NAT served as independent predictors of pCR (OR 1.119, 95% CI 1.031–1.213, *P* = 0.007; OR: 2.169, 95% CI 1.336–3.519, *P* = 0.002) (Table 5). Following this, Delta-lymphocyte subsets and Delta-LMR with significant differences in univariate analysis were included as independent variables in a multivariate logistic regression model. After adjusting for the effect of Ki-67 level, the results showed that dCD3 + T cells, dCD8 + T cells, and dLMR were also independent predictors of pCR (OR 1.101, 95% CI 1.019–1.191, *P* = 0.015; OR 0.892, 95% CI 0.821–0.968, *P* = 0.006; OR 1.960, 95% CI 1.004–3.827, *P* = 0.049) (Table 6).

**Table 5:** Comparison of independent predictive factors in lymphocyte subsets and LMR before NAT for pCR.

Indicators	B	SR	Wald	*P*-value	OR	OR 95% CI	
						Lower limit	Upper limit
CD3 + CD4 +	0.112	0.041	7.329	0.007	1.119	1.031	1.213
LMR	0.774	0.247	9.820	0.002	2.169	1.336	3.519
Ki-67 level	1.743	0.719	5.881	0.015	5.713	1.397	23.364

**Table 6:** Comparison of independent predictive factors in Delta-lymphocyte subsets and Delta-LMR for pCR.

Indicators	B	SR	Wald	*P*-value	OR	OR 95% CI	
						Lower limit	Upper limit
dCD3 +	0.096	0.040	5.881	0.015	1.101	1.019	1.191
dCD3 + CD8 +	− 0.115	0.042	7.408	0.006	0.892	0.821	0.968
dLMR	0.673	0.341	3.890	0.049	1.960	1.004	3.827
Ki-67 level	1.453	0.705	4.249	0.039	4.278	1.074	17.037

### ROC curve reveals the predictive value of lymphocyte subsets and LMR for NAT efficacy

**Figure 1:**
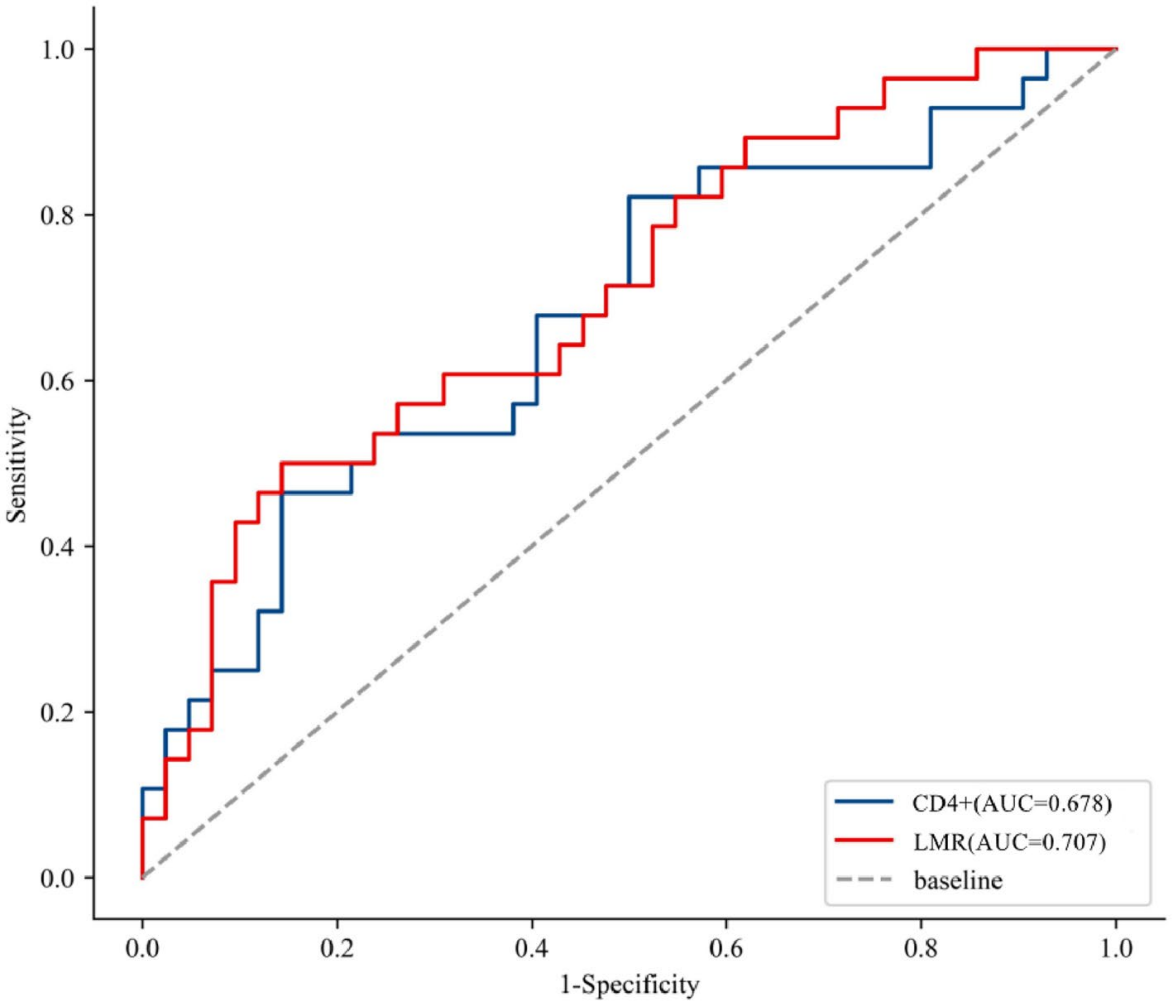
The ROC curve graph of lymphocyte subsets and LMR before NAT in pCR and non-pCR groups.

**Figure 2:**
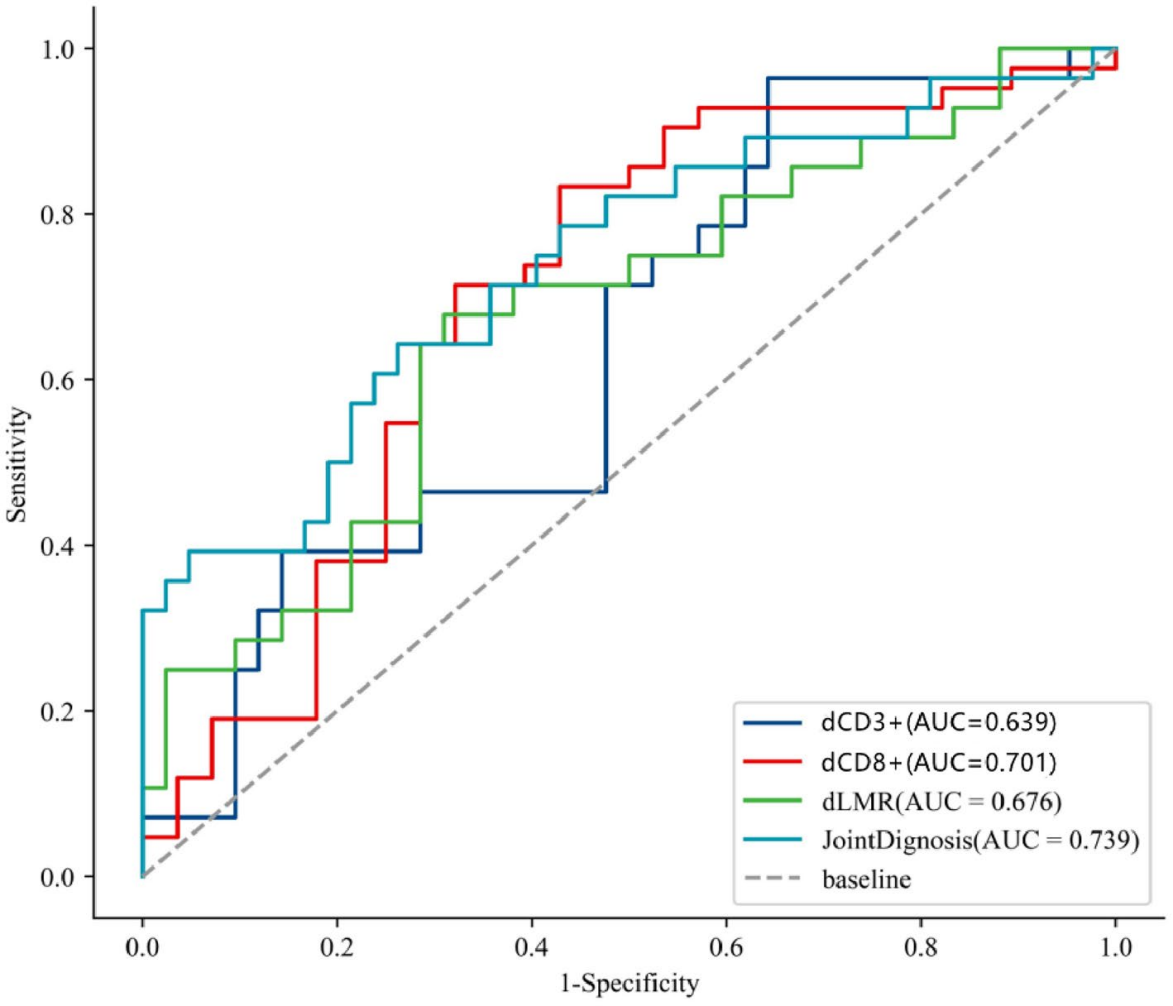
The ROC curve graph of Delta-lymphocyte subsets and Delta-LMR in pCR and non-pCR groups. JointDiagnosis stands for combination of dCD3 + T cells and dCD4 + T cells.

ROC curve analysis showed that the AUCs of CD4 + T cells and LMR before NAT were 0.678 (95% CI 0.548–0.808) and 0.707 (95% CI 0.583–0.831), with sensitivities of 46.4% and 50.0%, specificities of 85.7 and 85.6%, respectively. The AUCs of dCD3 + T cells, dCD8 + T cells, and dLMR were 0.639 (95% CI: 0.507–0.770), 0.701 (95% CI: 0.568–0.833), 0.676 (95% CI 0.546–0.806), respectively. Their sensitivities were 71.4, 83.3, 67.9, and specificities were 52.4, 57.1, 69.0%, respectively (Table 7; Fig. 1). When the lymphocyte subsets indicators dCD3 + T cells and dCD8 + T cells were used together for diagnosis, the AUC was 0.739 (95% CI: 0.617–0.861), with a sensitivity of 64.3% and a specificity of 73.8% (Table 8; Fig. 2). Hence, CD4 + T cells, LMR before NAT, and dCD3 + T cells, dCD8 + T cells, dLMR were significantly correlated with pCR and possess predictive value. Moreover, utilizing lymphocyte subset indicators in combination further improved their predictive capability.

**Table 7:** ROC analysis of lymphocyte subsets and LMR before NAT in predicting NAT efficacy.

Indicators	AUC	Sensitivity	Specificity	Yoden Index	Threshold
CD3 + CD4 +	0.678	0.464	0.857	0.321	46.890
LMR	0.707	0.500	0.856	0.357	4.684

**Table 8:** ROC analysis of Delta-lymphocyte subsets and Delta-LMR in predicting NAT efficacy.

Indicators	AUC	Sensitivity	Specificity	Yoden Index	Threshold
dCD3 +	0.639	0.714	0.524	0.238	6.155
dCD3 + CD8 +	0.701	0.833	0.571	0.405	− 0.020
dLMR	0.676	0.679	0.690	0.369	− 1.075
Joint diagnosis	0.739	0.643	0.738	0.381	0.477

## Discussion

In recent years, research has shown that the occurrence, progression, and metastasis of tumors are closely related to the immune status and inflammatory response in the tumor host^16^. A decrease in the body’s immune surveillance function and the immune cytotoxic function allows abnormal proliferative cells and tumor cells to achieve immune escape, promoting the formation and progression of malignant tumors, including breast cancer^17,18^. The most direct indicator of the body’s immune ability is the number of lymphocytes. According to different surface markers and differentiation antigens, lymphocytes are divided into different subsets. CD3 antigen is found on mature T lymphocytes, making it a general indicator for T lymphocytes and strength of cellular immune response. Mature T lymphocytes further express CD4 or CD8 antigens on their surface. CD4 + T cells can directly secrete cytokines such as IFN-α, TNF-γ, IL-2, which have anti-tumor effects, and also activate immune effector cells such as CD8 + T cells and NK cells to indirectly exert anti-tumor effects^19^. CD8 + T cells not only release granzyme and perforin to kill tumor cells, but also differentiate into cytotoxic T lymphocytes (CTL) to directly play an anti-tumor effect. Under normal conditions, most of the interior of CD8 + T cells are made up of CTL, and a few are suppressor T lymphocytes (Ts), which can inhibit the body’s immune response^20^. The dynamic balance within the T cell subsets is reflected by the CD4 + /CD8 + ratio. A decrease in this ratio indicates that the lymphatic system inside the patient is in a disordered state, and the anti-tumor ability of the immune system is reduced^21^. CD3 + CD4 − CD8 − T cells are a rare T lymphocyte subset that exerts anti-tumor effects through mechanisms such as the Fas/FasL pathway, cytokine secretion, and expression of tumor necrosis factor-associated apoptosis-inducing ligand (TRAIL)^22,23^. CD3 + CD4 + CD8 + T cells are a small subset of lymphocytes that has received relatively little research attention. Currently, there are two mainstream views regarding the role of CD3 + CD4 + CD8 + T cells. One view is that CD3 + CD4 + CD8 + T cells are highly cytotoxic as they express numerous cytotoxic-related immune molecules such as CRTAM, CD244, granzyme B, perforin, and IFN-γ^24^; Another view is that CD3 + CD4 + CD8 + T cells have immunosuppressive properties as they express IL-10 and IFN-γ, which reduce T-cell proliferation^25,26^. CD19 + B cells are a type of inhibitory B lymphocyte that can increase the expression of regulatory T cells to inhibit the activation of CD4 + T cells and promote immune escape in tumors^27^. NK cells are non-specific immune cells derived from bone marrow large granular lymphocytes. They have the ability to directly kill tumor cells through nonspecific killing without specific sensitizing antigens for activation^28^. NKT cells are unique immune cells that have both NK cells functions and T cell characteristics. They can recognize glycolipid antigens presented by CD1d molecules on tumor cells and exert tumor-killing effects by relying on CD95/CD178 molecules^29^. In this study, breast cancer patients had significantly reduced levels of CD3 + T cells, CD4 + T cells, CD4 + /CD8 + ratio, and NK cells, confirming that the transition of a tumor from benign to malignant can lead to significant immunosuppression. This suggests that the specific and non-specific killing effects of immune cells are impaired in these patients. It is possible that tumor cells inhibit lymphocyte differentiation and proliferation by secreting immunosuppressive factors such as TGF-β and IL-10, which leads to an imbalance in lymphocyte subsets^30^. Furthermore, the decrease in CD4 + /CD8 + ratio observed in breast cancer patients further revealed the presence of an immunological disorder. Previous studies have demonstrated that low CD4 + /CD8 + ratio is a crucial indicator of the severity and poor prognosis of malignant tumors, with a lower ratio indicating a higher risk of tumor metastasis and recurrence^31^. However, the occurrence of malignant tumors is not determined by a single factor. As early as Rudolf Virchow’s research, it was found that there were white blood cells in tumor tissue. When the body’s repair function was disordered due to a tumor, it stimulated the body to produce an inflammatory response that created a favorable microenvironment for tumor cells, accelerating their growth. This initialized the hypothesis of “relationship between inflammation and cancer”^32^. Monocytes, as a type of inflammatory cells, not only secrete inflammatory factors such as IL-1, IL-6, IL-10, and TNF-α to promote tumor microangiogenesis, but also assist in the adhesion of tumor cells to endothelial cells, thereby promoting breast cancer metastasis^33^. Monocytes are recruited to the peritumor tissue and differentiate into tumor associated macrophages (TAMs). TAMs contribute to tumor cells migration and invasion by reshaping the extracellular matrix and releasing mediators like VEGF, PDGF, and EGF. These mediators induce a hypoxic microenvironment and facilitate the formation of tumor microvessels^34,35^. TAMs also express IDO and IL-10 to play immunosuppressive roles^36^. In this study, we found that breast cancer patients had significantly lower LMR compared to benign breast tumor individuals, indicating that chronic inflammation might be a risk factor for breast cancer. Previous studies have shown that a lower LMR is indicative of a higher tumor burden in patients. Additionally, a low LMR prior to treatment is significantly linked to unfavorable DFS and overall survival (OS)^37^. Therefore, the immune system and inflammatory response are crucial factors in the occurrence and progression of breast cancer. Tumor cells originate from genetic mutations, leading to disruption in the body’s repair mechanisms that stimulates chronic inflammation. The presence of tumor cells also causes tumor-associated immunosuppression. Additionally, abnormality in immune function reduces the elimination of chronic inflammation. Ultimately, the prolonged presence of immunosuppression and chronic inflammation promotes tumor progression in turn, which forms a vicious circle. A deeper comprehension of the intricate relationships between these three factors is currently a hot topic in breast cancer research. And understanding these interactions can lay the groundwork for breaking the vicious cycle, consequently leading to more precise and effective treatment options for breast cancer in the future.

Currently, NAT is the preferred initial treatment for locally advanced breast cancer (LABC). Recent studies indicated that attaining a pCR after NAT leads to a more favorable prognosis and increased survival rates compared to those without pCR^38,39^. Therefore, early prediction of NAT efficacy is crucial in assessing drug sensitivity, selecting subsequent treatment regimens, and improving overall survival quality. Previous research has shown that effective treatment can rectify immune disorders, restore immune function, and alleviate chronic inflammation in patients with malignant tumors, despite their suppressed immune function^40^. In this study, 70 breast cancer patients underwent NAT. Among them, 28 achieved pCR, while 42 did not. Prior to NAT, notable disparities in lymphocyte subsets were observed between the two groups. Individuals in the pCR group exhibited significantly elevated levels of CD4 + T cells and NK cells in comparison to the non-pCR group. A possible mechanism is that the higher levels of T cells before NAT can help induce anti-tumor immunity during chemotherapy. Chemotherapy can stimulate the body’s immune response to the tumor. A more robust immune system function prior to chemotherapy is associated with a more intense immune response. This, in turn, enhances the effectiveness of chemotherapeutic drugs^41,42^. Denkert et al.^43^ discovered that the presence of tumor-infiltrating lymphocytes (TILs) is associated with increased response rates in breast cancer patients receiving NAT, and the enrichment of TILs can improve chemotherapy sensitivity. CD4 + T cells are one of the major components of TILs^44^. In addition, Verma et al.^45^ found that elevated NK cells in peripheral blood may be associated with favorable therapeutic effects of chemotherapy on breast cancer patients, possibly through mechanisms such as promoting NKG2D expression and the presence of Granzyme B( +)/perforin( +) cells. This study also discovered a positive correlation between high LMR prior to NAT and increased pCR rates. This suggests that individuals with lower levels of chronic inflammation before NAT are more likely to exhibit a heightened pathological response rate. Previous studies have found that TAMs can predict the sensitivity of radiotherapy and chemotherapy. This is because TAMs can not only release chemotherapy resistance factors, making breast cancer cells insensitive to chemotherapy, but also exhibit increased release of VEGF under radiation exposure, thus reducing radiotherapy sensitivity^46^. Ni et al.^47^ analyzed 542 LABC patients and found that high LMR before NAT is a significant favorable factor for NAT response and prognosis. This study’s multivariate analysis further demonstrated that high levels of CD4 + T cells and LMR before NAT are independent predictors of better pathological response rates. Additionally, high Ki-67 expression predicts tumor cell proliferation activity, and chemotherapeutic agents have stronger killing effects on proliferating cells. Consistent with previous studies, we found a correlation between high Ki-67 expression and a high pCR rate, suggesting that Ki-67 is also a factor related to NAT efficacy^48^.

Chemotherapy, as a systemic treatment approach, exhibits cytotoxic effects on tumor cells. However, due to the limitations in drug selectivity, it inevitably leads to collateral damage to the normal immune system of the body. Nevertheless, once the tumor cells are effectively eliminated by the chemotherapy drugs, their inhibitory effect on the immune system diminishes accordingly. Concurrently, the inflammatory stimulation within the tumor microenvironment also decreases^49,50^. Therefore, the role of chemotherapy in the immune status and chronic inflammation of the body is worth further research and exploration. Different from previous studies, our research also focuses on the changes in lymphocyte subsets and LMR before and after NAT, denoted as Delta-lymphocyte subsets and Delta-LMR. These dynamic quantitative indicators allow for a clearer representation of the changes brought about by NAT, while avoiding biases caused by inconsistent baseline characteristics among patients. The findings of this study found significant differences in the levels of dCD3 + T cells, dCD4 + T cells, dCD8 + T cells, dCD4 + /CD8 + ratio, and dLMR between pCR and non-pCR patients after NAT. To be more specific, the pCR group showed a significant increase in CD3 + T cells, CD4 + T cells, and CD4 + /CD8 + ratio compared to the non-pCR group. This indicate that after effective treatment, the tumor burden in the patients is significantly alleviated. As a result, the suppressive effect of tumor cells on the immune system is weakened, leading to a gradual recovery of immune function and normal production of CD3 + T cells and CD4 + T cells. In contrast, the non-pCR group exhibited a decrease in CD4 + /CD8 + ratio after treatment. This suggests that for chemotherapy-insensitive patients, not only do chemotherapy drugs damage the immune system but their tumor burden is not effectively reduced. During tumor progression, immune suppressive factors continue to be released, disrupting the homeostasis within lymphocyte subsets. Consequently, there is an increased dominance of lymphocytes with suppressive effects, causing a shift in the CD4 internal Th1/Th2 balance towards Th2. Additionally, there are an enhancement of activity and an increase in the number of Ts cells characterized by the CD8 + phenotype^51,52^. Simultaneously, the increased Ts cells have a regulatory inhibitory effect on CD4 + T cells, hindering their expression and maturation. As a result, the surface expression of CD4 antigen on lymphocytes decreases and the reactivity of CD8 + T cells increases. However, during this process, the body is in an immunosuppressed state, and the increased number of CD8 + T cells is dominated by Ts cells, further exacerbating the suppressive effects on CD4 + T cells^53,54^. Therefore, an increase in CD8 + T cells proportion and a decrease in CD4 + /CD8 + ratio were observed after NAT among patients with high tumor burden in the non-pCR group. During this period, the body experiences severe immune disorders, hindering its ability to effectively exert anti-tumor effects. Furthermore, this study revealed that following NAT, there was a decline in LMR for both the pCR and non-pCR groups. However, the decrease in LMR was notably greater in the non-pCR group than in the pCR group, and this difference was statistically significant. One possible reason is that chemotherapy drugs such as taxanes have been found to increase serum levels of IL-2, IL-6, GM-CSF, IFN-α, TNF-γ, and PGE2, these factors not only stimulate the proliferation of immune effector cells to exert anti-tumor effects but also trigger an inflammatory response in the body. This leads to the proliferation of monocytes and macrophages, which further intensify the inflammatory reaction by engulfing or phagocytosing antigenic substances^55,56^, resulting in a decrease in post-treatment LMR. However, patients who achieved pCR experienced immune function restoration, enhancing the inhibitory effect on this exacerbated inflammatory response. As a result, although the LMR might decrease in these patients, the extent of decrease was much lower compared to non-pCR patients. Our additional ROC curve analysis showed that dCD3 + T cells, dCD8 + T cells, and dLMR had good predictive value for treatment efficacy. Early identification of patients with poor therapeutic effects is key to subsequent treatment decisions. For these patients, their immune function remains suppressed and chronic inflammation is constantly activated, which further promotes tumor proliferation. As a result, even after completing the scheduled NAT cycle, they may fail to achieve ideal pathological complete remission.

Past research demonstrated a close correlation between the occurrence of tumors and the immune status of the organism, with immune cells participating in various pathways involved in the initiation and progression of tumors. In previous studies on patients with malignant neoplasms such as lung cancer, esophageal cancer, colorectal cancer, and gastric cancer, researchers have detected decreased levels of CD3 + T cells, CD4 + T cells , NK cells, and CD4 + /CD8 + ratio in peripheral blood^4,57–60^. The commonality among these findings reveals a prevalent state of immunosuppression in patients with malignant tumors, suggesting that changes in immune cell populations may be intimately associated with tumor burden in the body. This study examines the correlation between immune status, chronic inflammation, and NAT in breast cancer patients. It demonstrates the significance of lymphocyte subsets and LMR in forecasting the effectiveness of NAT in breast cancer. The aim is to provide clinicians with new prediction methods and reference criteria for NAT efficacy, which can improve prediction accuracy and provide more support for precise breast cancer treatment. Early identification of patients with poor treatment efficacy and timely adjustment of their treatment plan may improve the overall prognosis. However, this study still has some limitations. Firstly, it is a small-sample, single-center study. To confirm the conclusions drawn in this study, there is a need for large-sample, multi-center clinical studies. Secondly, venous blood samples were collected and analyzed only before NAT and prior to surgery in this study. However, immune damage and chronic inflammation are persistent processes, and dynamic monitoring of venous blood may provide a clearer understanding of these chronic processes. Additionally, long-term follow-up allows for the collection of subsequent survival data from patients, enabling the investigation of the correlation between lymphocyte subsets, LMR, and long-term prognosis among patients receiving NAT.

## Data Availability

The data supporting the findings of this study are available from the corresponding author upon reasonable request.
